# Return of individual genomic research results: are laws and policies keeping step?

**DOI:** 10.1038/s41431-018-0311-3

**Published:** 2019-01-08

**Authors:** Adrian Thorogood, Gratien Dalpé, Bartha Maria Knoppers

**Affiliations:** Centre of Genomics and Policy, McGill University, 740 avenue Dr. Penfield, suite 5200, Montréal, QC H3A 0G1 Canada

**Keywords:** Genetics research, Translational research

## Abstract

Efforts are underway to harmonise the return of individual results and incidental findings from whole genome sequencing (WGS) across research contexts and countries. We reviewed international, regional and national laws and policies applying to return across 20 countries to identify areas of convergence and divergence. Discrepancies between laws and policies are most problematic where they cannot be reconciled through harmonisation of project-level governance. Rules for the return of results apply at different levels in different jurisdictions (e.g., human subjects research, biobanks, clinical trials, genomic sequencing, and genetic/personal data), complicating comparison. A particular concern for harmonisation are the (often contradictory) rules about when results must, should, may, or must not be returned. Adding confusion are different thresholds for utility (medical, familial, reproductive, and/or personal). The importance of respecting individual choices to know or not know is widely recognised, though some norms emphasise respect for personal preferences. Another troubling observation is that requirements for data quality, variant assessment, and the effective communication of results are evolving in uneven ways. There is a growing gap between researchers with the expertise, infrastructure, and resources to meet these requirements and those without, threatening international collaboration. Best practices for the return of individual genomic results are sorely needed to inform not only the ethical return of results, but also future legislative and policy efforts.

## Introduction

The return of individual results and incidental findings in research is fraught with ethical and practical complexity, especially where participants undergo whole genome sequencing (WGS). Numerous law and policy making initiatives have attempted to clarify researcher responsibilities (see Table 1), only to replace ethical uncertainty with compliance uncertainty [1, 2]. As our review will demonstrate, this compliance uncertainty stems from inherent difficulties in articulating “bright line” rules for the return of genomic results, as well as imprecise language (e.g., must/should/may). Legal and policy uncertainty is increased by inconsistent interpretations by research ethics committees (RECs) [3]. Some uncertainty is inescapable, given the heterogeneity of research areas incorporating genomics. Discrepancies between regulatory frameworks create additional complications for international research collaborations, prompting calls for harmonisation [4, 5]. Policies are also rapidly evolving in the clinical context. They aim to provide health professionals with guidance for handling incidental or secondary findings concerning patients and their families (see Table 2). While clinical policies may inspire research practices, this can also expand expertise, resource, and infrastructure requirements in unforeseen ways.

**Table 1:** Laws and policy approaches for the return of individual results from WGS in research

Jurisdiction	Domains	Approach (subject to individual consent unless otherwise indicated)
International Policies		
UNESCO, *Universal Declaration on the Human Genome and Human Rights*, 1997 [1]	Genetic research	• Right to know/not to know
UNESCO, *International Declaration on Human Genetic Data*, 2003 [2]	Human genetics	• Right to decide about return of results • Right to access • MUST provide genetic counselling for results that may have a significant impact on a person’s health
UNESCO, *Universal Declaration on Bioethics and Human Rights*, 2005 [3]	Biomedicine	• Right to decide about the return of results
OECD, *Guidelines on Human Biobanks and Genetic Research Databases*, 2009 [4]	Biobanks and genetic databases	• MAY return validated results • MUST provide a plan for re-contact • SHOULD provide counselling by a trained professional
ISBER, *Best Practices for Repositories I: Collection, Storage and Retrieval of Human Biological Materials for Research*, 2012 [5]	Biobanks and genetic databases	• MUST have an ethics approved plan for handling the possibility of a return of results
WMA, *Declaration of Helsinki*, 2013 [6]	Human medical reasearch	• Right to be informed of (general) results of the study
WMA, *Declaration Of Taipei On Ethical Considerations Regarding Health Databases And Biobanks*, 2016 [7]	Biobanks and genetic databases	• MUST provide a plan for the return of results, including incidental findings.
CIOMS/WHO, *International Ethical Guidelines for Health-related Research Involving Humans*, 2016 [8]	Biomedical research	• MUST return life-saving information; data of immediate clinical utility involving a significant health problem; analytically valid, clinically significant and actionable results • DO NOT return results of “uncertain scientific validity or clinical significance” • MUST provide genetic counselling (as determined by a research ethics committee) • SHOULD provide a management plan (incidental findings, re-contact)
ICH, *Clinical Practice: Integrated Addendum to E6(R1)*, 2016 [9]	Clinical trials	• MUST return information to subjects concerning adverse events, including clinically significant laboratory values related to the trial • SHOULD provide adequate medical care to a subject for any adverse events
ICH, *Guideline on Genomic Sampling and Management of Genomic Data - E18*, 2017 [10]	Clinical trials (Biobanks and genetic databases)	• MAY return results of potential clinical significance and actionability • SHOULD provide a return plan describing clinical/genetic counselling, timing of return, by whom
Regional Laws		
Council of Europe, *Oviedo Convention on Human Rights and Biomedicine*, 1997 [11]	Biomedicine	• Right to know/not to know • MUST provide appropriate genetic counselling
Council of Europe, *Additional Protocol to the Convention on Human Rights and Biomedicine, concerning Biomedical Research*, 2005 and 2007 [12,13]	Biomedical research	• Right to know/not to know • MUST return results concerning current or future health or quality of life • MUST provide results within a framework of healthcare or counselling
EU, *General Data Protection Regulation*, 2018 [14]	Data protection	• Right of access to personal data
Regional Policies		
Council of Europe, *Recommendation (2006)4 of the Committee of Ministers to member states on research on biological materials of human origin and its Explanatory Memorandum, 2006* [15]	Biomedical research	• Right to know/not to know • MUST return any information collected about health of participants (including unexpected findings) • SHOULD provide a plan for disclosure of information from biological materials • SHOULD assess if research data anonymization is appropriate if there is a possibility of giving health-related feedback
National Legislation		
Costa Rica, *Biomedical Research Regulatory Law*, 2014 [16]	Biomedical research	• Right to access • MUST return results relevant to the health of the participant or about discovered diseases not part of the research process
Denmark, Executive Order no. 1464 of 2 December 2016 on Information and Consent to Participate in Health Research Projects, as well as Notification and Monitoring of Health Research Projects [17]	Health research	• MUST return significant health information
Estonia, *Human Genes Research Act*, 2001 [18]	Biobanks	• Right to know/not to know • Right to access data (except genealogies) by donor and physician (for treatment) • MUST provide genetic counselling to donors accessing their genetic data
Finland, *Biobank Act*, 2012 [19]	Biobanks	• Right to access information concerning health • SHOULD provide an account of result’s significance
France, *Code de la Santé Publique*, 2012 [20]	Genetic Information	• MUST return severe genetic disorders (anomalie génétique grave) and warn family members if participant refuses to do so.
Israel, *Genetic Information Law 5761-2000* [21]	Genetic information	• MUST provide genetic counselling
Italy, *General Authorisation No. 8/2012 for the Processing of Genetic Data*, 2014 [22]	Human Genetics	• MUST return results, including unexpected findings, with factual, direct benefits in terms of treatment, prevention and/or awareness of reproductive choices for the participant or someone in the same genetic line • MUST provide appropriate genetic counselling where necessary
Latvia, *Human Genome Research Law*, 2005 [23]	Genomic research	• Right to know/not to know • Right to access results • MAY return results related to the donor’s state of health to his or her physician if necessary for treatment (with consent)
Portugal, *Personal Genetic and Health Information Act*, 2005 [24]	Genetic Information (Biobanks)	• SHOULD provide the assistance of a doctor specialised in genetics
Spain, *Law 14/2007 of 3 July on Biomedical Research*, 2007 [25]	Biomedical research	• Right to know • MUST return results with health relevance • MUST return results if necessary to avoid serious damage to the health of the individual or his/her relatives, regardless of individual’s wishes • MUST provide genetic counselling for results indicative of a predisposition to a disease
Taiwan, *Human Subjects Research Act*, 2011 [26] *Human Biobank Management Act*, 2010 [27]	Human research Biobanks	• MUST provide statement of expected results from the research • DO NOT return of individual results [27] • Right to access (personal information only)
USA, *Common Rule*, 2017 [28]	Biomedical research	• MUST provide a statement whether clinically relevant research results, including individual research results, will be disclosed to subjects, and under what conditions; OR • MUST provide a statement that clinically relevant results, including individual research results, may not be disclosed to the subject if there is no plan to do so
National Policy		
Australia, National Health and Medical Research Council, *National Statement on Ethical Conduct in Human Research*, 2018 [29]	Human research	• MUST return findings that are of proven validity and of health significance to the participant • MAY return findings outside of the scope of the research • MAY review findings of a research project after the project has been completed • MUST prepare and follow an ethically defensible plan to manage the disclosure or non disclosure of genomic information of potential importance for the health of research participants; plan must be approved by research ethics board • MUST justify a plan to return findings during the project that are of proven validity but are not of health significance to the participant • SHOULD describe access to clinical advice and genetic counselling • SHOULD prepare to provide information to participants who later change their preferences and choose to receive information
*Australia, National Health and Medical Research Council, National Statement on Ethical Conduct in Human Research*, 2015 [30] (no longer current)	Human research	• MUST prepare and follow an ethically defensible plan to disclose or withhold that information • MUST consider in the plan: the clinical relevance of the research information; the types of genetic test used in the research; and the results of those tests • MAY offer participants the option of being notified of the existence of these results if the relevance of genetic information to participants’ health is not clear until after interim analysis • MAY offer genetic counselling when genetic results are important for the health of participants • MUST include a clear explanation of the difference between research and clinical testing in advice about the results of genetic research
Brazil, National Health Council (CNS), *Resolution 340/2004: Guidelines for Conduct and Ethical Analysis for Research Projects of the Special Thematic Area of Human Genetics*, 2004 [31]	Genetic research	• MUST have a proactive plan to handle expected results and their interpretation, genetic counselling, and consequences to subjects
Canada, Interagency Panel on Research Ethics, *Tri-Council Policy Statement: Ethical Conduct for Research Involving Humans*, 2014 [32]	Biomedical research	• MUST return any material incidental findings to the participant • MUST have an ethics-approved plan for managing return of genetic results • MAY offer genetic counselling to participants
Denmark, National Committee on Health Research Ethics, *Guidelines on Genomics Research*, 2018 [33]	Genomic research	• MUST return important health information to the participant (unless informed opt-out) • SHOULD *only* return genetic variants with a high penetration predisposing to a severe and treatable/curable/preventable disorder • SHOULD assess whether to return life-saving or disability preventing results to relatives (consent override) • MUST provide genetic counselling • MUST establish a committee of experts to assess incidental findings • MUST provide *pre-test* counselling for study of highly-penetrant genes
Germany, EURAT, *Cornerstones for an Ethically and Legally Informed Practice of Whole Genome Sequencing: Code of Conduct and Patient Consent Models*, 2015 (recommended by the German Research Foundation) [34]	Genomic sequencing	• MUST return medically relevant findings • DO NOT return additional findings that cannot be treated or prevented; changes with only a slight probability; carrier status.
German Society for Human Genetics, *Opinion of the German Society for Human Genetics on Genetic Incidental Findings in Diagnostics and Research*, 2013 [35]	Human genetics	• MUST return genetic characteristics that show a relevant risk for disease for which an effective therapy or effective preventative measures exist • MAY return genetic characteristics that show a relevant risk for a disease that cannot be treated at the time of diagnosis • DO NOT return genetic characteristics that increase the risk of the occurrence of a disease only slightly
India, Council of Medical Research, *National Ethical Guidelines For Biomedical and Health Research Involving Human Participants*, 2017 [36]	Biomedical research	• MUST return results that are actionable, leading to potential benefits of improving health outcomes • SHOULD offer genetic counselling • MAY offer ancillary medical care as a result of incidental findings from research • SHOULD develop a plan for return of incidental findings
Japan, Ministries of Education, Health, and Economy, *Ethical Guidelines for Human Genome/Gene Analysis Research*, 2008 [37]	Genetic research	• MUST disclose genetic information concerning participants upon request • MAY withhold disclosure of results (or part of the results) that would likely harm life, body or other interests of the participant • MUST provide explanations to donors for why results were not returned to them **•** MUST establish a plan for results expected from the research, the return of results and genetic counselling • MUST report treatable, life-threatening results for donor or relatives to director to assess if they should be returned
Singapore, Bioethics Advisory Committee, *Ethics Guidelines for Human Biomedical Research*, 2015 [38]	Biomedical research	• SHOULD return clinically significant results from genetic research • SHOULD refer to research ethics board if participant’s preferences for return are unknown • SHOULD provide a researcher or healthcare provider to advise on implications
UK, Human Tissue Authority, *E Research, Code of Practice and Standards*, 2017 [39]	Research	• SHOULD provide a plan to manage results (including incidental findings) where relevant
Medical Research Council and Wellcome Trust, *Framework on the feedback of health-related findings in research*, 2014 [40]	Health research	• SHOULD return health-related findings where benefits of return outweigh harms • MUST provide a clear, justifiable policy of return of results supported by evidence where possible • SHOULD develop a practical return procedure addressing how and when results will be identified, validity of findings, who will communicate results, how, and when feedback occurs
USA, National Academies of Science, *Returning Individual-Specific Research Results to Participants: Guidance for a New Research Paradigm*, 2018 [41]	Health Research	• SHOULD return results of high potential value to participants where feasible. • SHOULD NOT return results from labs without appropriate quality management systems. • SHOULD provide a plan for whether and how to return results in funding application, research protocol, and consent. • SHOULD incorporate participant needs, preferences, and values into decision-making process. • SHOULD have access to appropriate expert committees and communication resources. • SHOULD employ effective communication tools and strategies
MRCT Centre Return of Individual Results Workgroup, *Return of Individual Results to Participants: Recommendations Document*, 2017 [42]	Clinical trials	• MUST return urgent results (including incidental findings) that are outside of normal ranges and associated with an urgent need to return due to the potential consequence for diagnosis, treatment, or care of the individual • MAY return routine results and non-urgent incidental findings • MAY return exploratory results on a case-by-case basis • SHOULD consider genetic counselling for the return of pathogenic or likely pathogenic variants • SHOULD have an ethics approved return management plan
Industry Pharmacogenomics Working Group, *An Update to Returning Genetic Research Results to Individuals*, 2015 [43]	Clinical trials	• MAY return analytically and clinically valid results that have clinical utility • DO NOT return results with uncertain health, reproductive, or personal utility that do not provide likely net benefit to the contributor
Presidential Commission for the Study of Bioethical Issues, *Anticipate and Communicate: Ethical Management of Incidental and Secondary Findings in the Clinical, Research, and Direct-to-Consumer Contexts*, 2013 [44]	Clinical/research	• MAY consider personal utility as a factor to help categorise findings • SHOULD have an ethics-approved plan for disclosing and managing the return of incidental and secondary findings • SHOULD provide genetic counselling • MAY adopt a plan that includes looking for select, clinically significant and actionable secondary findings
National Heart, Lung, and Blood Institute Working Group, *Ethical and Practical Guidelines for Reporting Genetic Research Results to Study Participants*, 2010 [45]	Biomedical research	• MAY return individual genetic results that convey important health implications for the participant that are actionable and analytically valid, and with associated risks that are established and substantial • SHOULD return upon review by an expert panel and a research ethics board • SHOULD NOT oblige researchers to return results beyond study funding • MAY provide an exceptional procedure for researchers who want to return results not qualifying under the main return policy, with an assessment of the risk/benefit ratio of doing so

**Table 2:** Select policies for reporting individual findings from clinical genomic sequencing

Organisation	Policy	Return approach
ACMG	ACMG Policy Statement: Updated Recommendations Regarding Analysis and Reporting of Secondary Findings in Clinical Genome-scale Sequencing, 2015; Recommendations for Reporting of Secondary Findings in Clinical Exome and Genome Sequencing, 2017	• Routinely inspect a panel of 59 genes deemed medically actionable to detect pathogenic variants, which may be unrelated to reasons for testing (“secondary findings”) • Expert panel updates list of genes
ESHG	Whole-Genome Sequencing in Health Care: Recommendations of the European Society of Human Genetics, 2013	• Use a filter to avoid unsolicited findings, or findings that cannot be interpreted • Unsolicited findings indicative of serious health problems should in principle be returned.
CCMG	The Clinical Application of Genome-Wide Sequencing for Monogenic Diseases In Canada: Position Statement of the Canadian College of Medical Geneticists, 2015	• Use a filter to avoid incidental findings • Incidental findings that reveal risk for a highly penetrant condition medically actionable during childhood should be reported to the parents.

We reviewed laws and policies governing research involving genomic sequencing across 20 countries, in order to inform the content, possibilities, and limits of harmonising return of results policies internationally. Key definitions are provided in Box 1. Discrepancies between laws and policies are particularly problematic for international collaboration, as they cannot simply be reconciled by research governance (e.g., protocols and consents). Conflicts of law or policy may only be harmonised by amendment. Our comparison of regulatory instruments begins with a review of (A) general principles; and (B) human rights, namely the right to know, not to know, and the right to access. We then compare and contrast the instruments according to the following policy themes: (C) consent models for enabling autonomous choices; (D) return of results policy approaches (must, should, may, or do not return); and (E) process requirements and guidance for the return of results (establish a plan; variant assessment; and care/counselling). In our discussion, we consider the influence of clinical genomic test reporting guidelines on research; the expansion of “utility” to include familial, reproductive/carrier, and personal considerations; and concerns over data quality. For each theme, we identify the discrepancies most likely to result in a lack of “legal interoperability” between jurisdictions [6].

## Methodology

We reviewed international, regional, and national laws and policies applying to research involving genomic sequencing. We began with documents identified in three previous systematic reviews [7–9]. We then reviewed all documents in the most recent version of the comprehensive International Compilation of Human Research Standards [10]. Additionally, we searched the the PopGen Module of the HumGen database (a specialised database composed of norms applying to population biobanking) [11, 12] and the websites of the International Federation of Human Genetics Societies [13]. We included international, regional, and national norms that are current (i.e., in force), address the return of individual results, and apply to research involving genomic sequencing. Inclusion/exclusion of all documents and data extraction were carried out by both AT and GD. Disagreement between coders was resolved by a group discussion with all three authors. Data extraction categories listed in Table 1 and in the section headers below were determined inductively by all three authors. Database and in-text search strings included variations of terms including “individual research result” and “incidental finding”.^1^ Norms were included if an official version, official translation, or reliable peer-reviewed summary article was available in English or French. For policies, we included position papers, reports, guidelines, or consensus statements issued by governmental or non-governmental health organisations, bioethics committees, and professional associations. Our methodology has certain limitations. We did not systematically review laws or policies indirectly regulating return of results, such as those governing clinical devices, diagnostic tests, or medical professionals, though we discuss the influence of clinical norms in our discussion and Table 2. We excluded provincial/state level policies, and policies specific to national sequencing initiatives. Other than the European *General Data Protection Regulation* [14], we did not systematically review privacy or data protection norms, which include relevant individual access rights, consent requirements, and duties of confidentiality (which may restrict return of results to family members). We also did not explore norms specific to paediatric or incompetent adults contexts [7]. We limit the scope of our review to laws and policies, which can present the most serious limitations on harmonisation of research governance. Exploration of the diversity of return practices is left to future studies.

## Results

We identified laws and policies across 20 countries (see Table 1), variously governing biomedicine, health/biomedical research, biobanks and genetic databases, genetic testing/information, genetic/genomic research, and clinical trials. Some norms had return of results provisions for both a general domain (e.g., biomedical research), and a narrower domain (e.g., biobanking or genetic research), which may need to be read together (e.g., [20], [32]).

### General principles

We begin by introducing general principles guiding the return of results, citing some examples from policy documents: 1. The rights and interests of participants take precedence over the goals of research to generate new knowledge [6, art. 8-9;31, s IV.1]. 2. Researchers have some level of responsibility for the welfare of participants, and are responsible for their protection and for mitigating the risks of their participation in research [6]. 3. Consent to research is informed, voluntary and ongoing [6]. 4. Participants have a right to know information relevant to their health. The wishes of participants not to know health information, however, are also to be respected [1, art. 5c;2, art. 5;3, art. 6;11, art. 5]. 5. Participants’ privacy and the confidentiality of their identifiable information should be protected [8]. 6. Research policies on the return of results should be informed by participant and community engagement [8;41]. 7. Researchers have a duty to share general results of research with the public and with participants. This latter principle relates to transparency and public accountability, and could mistakenly be interpreted to encompass individual results, as well as aggregate results [6]. 8. Participants have the right to access their personal health data [14]. High-level principles are not sufficient to guide the return of results in all research contexts. There is often legitimate disagreement over the priority or application of these principles in return of results contexts, e.g., the intensity of researcher duties of care towards participants, or was constitutes meaningful engagement. These principles may even conflict in some cases, such as the researcher’s duty to mitigate harms and the participant’s right to know health information. Law and policy making efforts have responded by trying to articulate clearly defined *rules* for the return of results. As we discuss below, this may not be a helpful development, especially from the perspective of international research collaboration.

### Human rights

International and European human rights instruments articulate a “right to know” health information [1;11]. The right to know underpins policies requiring researchers to return clinically relevant information to participants (discussed below). These norms also recognise a “right not to know” health information [1;8;11]. The right not to know is explicitly stated in Estonia’s biobank law [18], Latvia’s genomic research law [23], and Italy’s genetic data privacy law [22]. This right is often implemented by offering participants choices about return of results (e.g., an opt-out). By contrast, under Spain’s biomedical research law, researchers must override a participant’s wish not to know in order to avoid serious harm to the participant or his or her relatives [25, art. 4.5]. Other norms, while respecting the right not to know in principle, express practical concerns about informed choices not to know in the context of genomics. Denmark’s genomic research guidelines, for example, require return of serious results unless there is an “unequivocal” wish not to know [33]. These guidelines also suggest that researchers should override wishes not to know for life-threatening or disability-preventing findings for relatives.

In some cases, the right to know is understood to include a “right to access”, i.e., a right to receive health information *upon request*. This right is distinct from a researcher duty to return results. The 2003 UNESCO *International Declaration on Human Genetic Data* stipulates that “[n]o one should be denied access to his or her own genetic data” [2, art. 13]. Moreover, the *European General Data Protection Regulation (GDPR)* provides individuals a right to access their personal data, though nations can establish exceptions in the research context [14, arts. 15, 89]. Finland’s 2012 biobank law provides donors a right to access “information concerning his or her health as determined based on a sample” [19, s 39]. Estonia’s biobank law provides a right to access, but excludes “genealogies” [18, art. 11(2)]. Japan’s genomic research policy includes an access right, but articulates a “therapeutic privilege” permitting researchers to withhold information that would seriously harm the participant [37, art. 23]. Similar to our discussion of general principles above, there is disagreement over how human rights apply to the return of results. As we discuss below, applying a right to know in genomic research can raise more questions than it resolves, namely what qualifies as relevant health information, who decides, and what qualifies as an unequivocal desire not to know?

### Consent

On a basic level, many international laws and policies encourage or require researchers to provide prospective participants with information about the project's return of results policy during the research consent process [5;7;8]. Researchers should establish an ethics-approved plan to return results or not and receive consent *thereto*. The contents of consent can naturally be derived from the plan. Sometimes, the return of results is conceptualised as posing a risk of economic or psychological harm, such as genetic discrimination. Numerous national laws [24;28] and policies [30;32;39;40;41] therefore recommend or require participants be informed of the return policy during the consent process before consenting. Denmark’s genomic research guidelines require pre-test counselling for the study of highly penetrant genes, and recommend consent include an estimate of the frequency of incidental findings [33]. The US Common Rule does not require a particular return approach, but requires consents to explain when clinically relevant research results will be disclosed or not, and under what conditions [28]. Other norms extend beyond providing information, and encourage or require researchers to offer participants a choice about receiving results (e.g., an opt-in or opt-out) [4;10;29;31;37;38;42–45]. The CIOMS/WHO guidelines go further and recommend offering participant tiered choices [8]. Australia’s health research guidelines even expect participants to have opportunities to *update* their preferences about the return of results [29, s 3.3.34]. Where research participants are informed that results may be returned, there is a countervailing concern that this will lead to “therapeutic misconception”. Participants may be harmed if they assume that receiving no results means nothing is wrong or, from a research study, believing they will receive personal benefit from the return of results. For this reason, the US Common Rule 2017 requires disclosure of any possibility that clinically relevant results might NOT be returned [28, § ll.116(D)(6)].

Such a diversity of consent policies raises challenges for research combining datasets across multiple jurisdictions. Prospective collaborations struggle to tailor consent clauses that satisfy multiple REBs. Studies combining existing sample or data collections across jurisdictions have difficulties to track and implement multiple policies and/or individual preferences for return. These logistical issues become more complex as datasets continue to be combined, shared and reused over time. In particular, approaches that favour informing participants that they *may* receive results may be difficult to harmonise with the US approach that favours informing participants that they *may not* receive results.

### Approaches to return

A number of laws and policies state rules concerning the return of results. These rules can be categorised as follows: (1) **must** return (2) **should** return (3) **may** return, or (4) **do not** return results to participants (Table 3). It bears noting that “must” and “should” rules are often conflated or confused. A “must” rule can still be found in norms without binding force. Rules requiring return of certain types of findings are most problematic for international harmonisation. Researchers might have to impose the same rule on their international collaborators, e.g., as a condition of material or data access agreements. Collaborators with limited resources or uncertain of what this responsibility entails could refuse such a strict term. The most common reasons for prohibiting or recommending against return are uncertainty over the scientific validity or utility of information for participants. Uncertainty often stems from resource, infrastructure or expertise limitations in a given research context. Prohibitions and requirements are most likely to conflict. A researcher prohibited to return results not generated in a clinical laboratory, for example, would not be able to comply with a collaborator’s requirement that certain types of results be returned. Researchers could face a double bind if prohibitions on returning certain results are also interpreted to apply to individual access requests. Under most “should” or “may” rules, return of results is conditional on one or more factors: analytical/clinical validity and clinical actionability, individual consent to receive results, and/or the availability of care or counselling resources. On the one hand, conditions can provide a loophole for researchers or their international collaborators not to return results. On the other hand, if conditions are interpreted as requirements of ethical research, they can effectively prohibit research or international collaboration where sufficient resources are not available.

**Table 3:** Policy and normative approaches for the return of results

**Must return**	
CIOMS/WHO	Life-saving information and data of immediate clinical utility involving a significant health problem [8]
COE	Significant results that concern the subject’s future health or quality of life [12,13]
ICH Topic E6(R2)	Information concerning adverse events, including clinically significant laboratory values related to the trial [9]
US MRCT	Urgent actionable results. Assess genomic results on a case-by-case basis [42]
Italy	Results with factual, direct benefits for treatment, prevention or reproductive choices [22]
Spain	Results of health relevance. Return overrides consent if it would avoid serious damage to health to participants or their families [25]
Canada	Material incidental findings that convey significant welfare implications [32]
France	Severe genetic disorders and warn family members if participant refuses to do so [20]
India	Actionable and have potential health benefits [36]
Germany	Relevant genetic risks for disease for which an effective therapy or preventative measure exists [35]
Australia	Findings that are of proven validity and of health significance [29]
Denmark	Important health information [33]
India	Results that are actionable and leading to potential health outcomes [36]
Costa Rica	Results relevant to the health of the participant or about discovered diseases not part of the research process [16]
**Should return**	
ICH Topic E6(R2)	Inform subject when medical care is needed for intercurrent illnesses [9]
US NASEM	Individual research results of high potential value to participants where feasible [41]
Denmark	Genetic variants with a high penetration predisposing to a severe and treatable/curable/preventable disorder [33]
Singapore	Clinically significant results from genetic research [38]
UK	Health-related findings where benefits of return outweigh harms [40]
**May return**	
OECD	Validated results [4]
ICH E-18	Results of potential clinical significance and actionability [10]
US Common Rule	Consent guidance only [28]
UK Human Tissue Act	Related guidance documents explore the conditions of responsible return or not [39]
Australia	Incidental findings [29]; results if the relevance of genetic information to participants’ health is not clear until after interim analysis [30]
Germany	Results that show a relevant risk for a disease that cannot be treated at the time of diagnosis [35]
**Do not return**	
CIOMS/WHO	*Prohibits* return of results not scientifically valid and/or clinically significant [8]
Taiwan (law applies to national biobank)	*Prohibits* return of individual results [27]
Germany	*Prohibits* return of genetic characteristics that only slightly increase disease risk, cannot be treated or prevented, or carrier status [34]
Denmark	*Recommends* only highly penetrant variants for severe and actionable disorders be returned [33]
US NASEM	*Recommends* only results from a laboratory with clinical accreditation or an appropriate quality management system be returned [41]
NHLBIWG	*Recommends* not obliging researchers to return results beyond study funding [45]

*Note*: the return policy is subordinate to the consent of the individual to receive results (or not) unless otherwise indicated

### Process requirements and guidance

Many international and national guidelines recommend that research projects establish a plan for handling individual results, for review in advance by a REC [8 G11-12; 10, art. 5-6;30, s, 3.5.1-3.5.2;31, s III.3;32, art. 3.4]. Where a plan has been established in advance, the possibility (or not) of a return of individual results can also be explained in the consent [8, G11-12; 28; 30, s 3.5.2; 32, art. 3.4]. A dimension not often considered in such plans is sample or data sharing. i.e., whether or not collaborators are required to adopt the return plan as a condition of access. Furthermore, international researchers without comparable resources, infrastructure or expertise may be precluded from collaboration.

Additional procedural inconsistencies could complicate international collaboration. Norms differ as to whether retrievable results should first be assessed by a physician, an REB [37;38;41;42;44;45], or an interdisciplinary expert committee (e.g., Denmark [33]). When results are returned, the provision of clinical care or genetic counselling may be required. UNESCO requires counselling for genetic results of “significant impact” [2, art. 11]. CIOMS/WHO recommends that an REB should consider if counselling should be provided [8, G11-12]. Both the Council of Europe’s OVIEDO Convention and its additional protocols and recommendations all require that return happen in a proper healthcare or counselling framework [11, art. 12;12, art. 27;13, art. 27]. Spain’s health research law, Estonia’s biobank law, and Israel’s genetic information law all require counselling upon return or access to results [18, § 11(4);21, art. 14(c);25, art. 9.3]. Finland’s Biobank Act requires an “account” of the result’s significance [19, s 39]. Italy’s law requires counselling “where necessary” [22, art. 9]. Portugal’s research law recommends a genetics specialist be involved [24, art. 19]. A number of policies also recommend that counselling should be provided, or at least be considered in the return of results plan [2;4;8;10;11;29–31;33;36;37;42;44;45]. The US 2018 NASEM report recommends that research institutions, funders and researchers ensure training, resources, infrastructure, policies and procedures are in place to ensure the quality of and effective communication of individual research results, including tools to promote participant understanding (e.g., synoptic information, means of follow-up) [41, Rec 4, 8, 10]. Dilemmas arise where researchers want to return a result through an international collaborator, but the collaborator does not provide a comparable level of support to ensure the communication is effective and ethical. The problem with procedural requirements is they assume particular resources are in place. These assumptions may not hold where collaborations extend across sectors or jurisdictions.

## Discussion

### The influence of the clinical context

The laws and policies governing the return of results from genomic sequencing in research are increasingly influenced by professional societies recommending clinical testing reporting standards (see Table 2 for a summary of select policies). Research norms now emphasise clinical concepts including ‘analytical validity’, ‘clinical significance’ and ‘actionability’ criteria [7]. An important insight from clinical genomics is recognition that the degree to which sequencing generates incidental or secondary findings “is a function of technical and analytic choices” [14]. Concerned that genomic sequencing will inundate clinicians and patients with results of uncertain validity, significance and actionability, clinical norms generally recommend a gene panel approach. There are several approaches for selecting the targets for a gene panel, ranging from the inclusion of all possible genes involved in a disorder to a more conservative selection of genes with strong evidence of linkage, clinical significance and high actionability, and/or limiting incidental findings where interpretation resources are limited. The European Society of Human Genetics (ESHG) [15], Canadian College of Medical Geneticists (CCMG) [16], and Belgian WES/WGS guidelines recommend filtering out incidental findings, except in special cases [17]. The influential approach of the American College of Medical Genetics (ACMG) recommends that a standard gene panel of “secondary findings” be actively analysed for all genomic sequencing. Secondary findings are those not known to be associated with the patient’s condition but that may have compelling “genetic, biological, and pathological features that implicate them in a patient’s underlying phenotype” [18, 19] or that present “urgent and serious implications for…health or welfare” [20]. The ACMG “list” is periodically reviewed and updated by an expert committee [18, 21].

The popularity of gene panel approaches may raise expectations for researchers to “hunt” for secondary findings. Critics already point out that the routine search for extra genes in clinical contexts is not only expensive, but is also “opportunistic screening” without a sufficient public health basis [22]. With the exception of translational genomics projects seeking to assess the value of using WGS in the clinic, an active search for secondary findings will not be appropriate in research contexts lacking the expertise, infrastructure and resources of clinical organisations.

### Familial, reproductive, or personal utility

The norms reviewed reflect expansion of the concept of utility to the health of family members, carrier status/reproductive decision making, and personal utility. Spain’s law requires return of information of health relevance to participants or relatives; return to avoid serious damage to family members overrides participant consent [25, art. 4.7]. France requires physicians via an intermediary to report potentially serious genetic test results to family members if the individual refuses to do so [20, art. R1131-20-2]. Italy’s genetic information law authorises the disclosure of individual results that may prevent “health from being jeopardised” for individuals “in the same genetic line” as the participant [22, art. 9]. Italy’s law also includes consideration of results of reproductive importance [22, art. 9]. Carrier status may include information about autosomal recessive disease that pass on to future children and X-linked disease for women carriers. Such results may also be important for relatives [22, art. 9]. The 2016 European Society of Human Genetics guidelines for diagnostic next-generation sequencing state that “genetic counselling should be provided for opt-in/opt-out of carrier status for unrelated and secondary findings” [23]. By contrast, Germany’s genomic sequencing guidelines recommend that carrier status should not be returned [34].

A number of policies broaden the scope of return to findings with personal utility, information that can help “decisions, actions or self-understanding which are personal in nature” [42]. Canada’s research guidelines require return of “material” incidental findings with “welfare implications” for the research subject [32, art. 3.4]. The *US Presidential Commission for the Study of Bioethical Issues* of 2013 recommends that professional organisations develop guidelines for the return of incidental and secondary results that take into account the personal utility of results [44]. In 2017, the Harvard-led MRCT called for researchers to take into account not only the clinical significance but also the “personal utility” of findings [42]. Yet, the USA 2018 NASEM report warns investigators against making assumptions about what people value, and outlines strategies for incorporating participant needs, preferences, and values into decisions about the return of individual results (e.g., consultating community and participant groups). [41, Recommendation 5].

There is a shift towards reporting individual findings that, in addition to risks of health or reproductive importance, have “personal utility to the contributor” [24]. Personal utility can be grouped under 3 main categories: affective, cognitive, and behavioural [25]. For instance, personal utility can be seen as the emotional state of individuals in situations when they have to cope with findings indicating health risk (*affective*); how individuals value the genetic information from test results disclosed to them and how they conceptualise genetics in their disease aetiology (*cognitive*); how individuals plan their future in view of recently disclosed genetic results, particularly with the subject of reproduction and, how they handle communications with family members on the subject of shared genetic traits (*behavioural*). Affected persons, as opposed to health professionals, tend to see utility beyond improved healthcare outcomes, including life and family planning, relief and justification, self-knowledge and self-determination [26]. While the concept of benefit is being expanded, the results “must still meet some objective criteria to provide a reasonable rationale for action.” [26]

On one hand, expansion of the meaning of utility may be welcome from a beneficence point of view. On the other hand, this expansion complicates interpretation and assessment of results, perhaps at significant expense for researchers. We also see that this expansion is uneven across jurisdictions. This generates discrepancies across jurisdictions that may take international collaborations significant time and effort to clarify.

### Quality/validity

A major concern for sequencing and return of individual results in the research context is data quality. There is significant debate about how to handle the questionable analytic validity of genomic sequencing and clinical validity of research findings. This is an area experiencing rapid international divergence. Particularly problematic for international collaboration are prohibitions on the return of results not generated in clinically accredited laboratories (the US position) [42]. The USA 2018 NASEM report, while highlighting the importance of quality, recommends a more flexible approach including certification for or case-by-case quality assessment of research laboratories [41, Recommendation 3]. Germany’s genetic testing law requires validation of research results in an approved genetic laboratory [27]. Strict quality requirements preclude return of results and increase the costs of research but constitute an ethical imperative that is often forgotten, especially considering the overlap between research and clinic. Tying quality requirements to national laboratory accreditation processes, however, may preclude the participation of foreign laboratories in international collaborations.

## Conclusion

Our comparative study of approaches to the return of individual results from whole genome sequencing in research— covering international, regional (Europe), and national laws and policies (**20** countries)—reveals an impressive diversity of positions. Explanations for this heterogeneity may include: different cultural views about participant and familial needs, different national health care systems and research sector resource constraints, lack of sensitivity to differences between the terms “must” as opposed to “should” or “may” (when articulating a positive obligation to act with potential legal consequences), or the conflation of research and clinical contexts. In each section, we have identified areas where law and policy discrepancies are most likely to conflict in ways that hinder international collaboration. It will not always be possible for collaborations to overcome these discrepancies by harmonising their return of results policies and tools (e.g., consents). Law or policy reform may be required. As a stop gap solution, international collaborations may want to design return of results processes that still allow for return decisions to be made locally. We did not study variation between individual project-level return policies, which would presumably also reflect different research contexts (e.g., epidemiological vs. fundamental vs. clinical research), or different local REC interpretations. This variation can also complicate international collaboration and merits further study. We conclude with a rough sketch of best practices to guide future efforts to harmonise laws, policies, and protocols:

- Establish a plan for the return of individual results for prior REC approval,
- Inform participants of this approach,
- Clarify the choices available to participants,
- Use a qualified genetic testing laboratory for validation,
- Explain any limitations on possible return (nature of WGS results to be returned, by whom, when, and over what period of time),
- Mention possible familial, reproductive/carrier and insurance implications, and
- Provide a process for expert determination of “actionability” if return is foreseen.

In our view, respecting individual choice—informed by the broader familial and social implications of WGS—is primordial but must be tempered and balanced by the needs of efficient, economic, and ethical research and health care. This common goal illustrates the “right of everyone to share in scientific advancement and its benefits” (art. 27 Universal Declaration of Human Rights, 1948). A return of results policy should serve to support the realisation of this right. To keep step, harmonised, systematic approaches must be developed to accelerate the transmission of lessons learnt, clarify approaches, improve individual outcomes, and ensure the efficacy and sustainability of health care systems.

Box 1 Definitions

- **Whole genome sequencing**: the method used to determine the complete DNA sequence of an individual’s genome
- **Whole exome sequencing**: the method used to determine the nucleotide sequence primarily of the protein-coding regions of an individual’s genome and related sequences
- **Primary finding:** a finding relevant to the explanation, main diagnosis or treatment of the disease obtained in the context of clinical care
- **Secondary finding**: an additional, looked-for finding, that is not pertinent to the main condition
- **Incidental finding**: a finding discovered in the course of research or testing concerning beyond the aims of the research. Also referred to as serendipitous, unanticipated, unsolicited, or off-target findings
- **Individual research result**: individual finding related to the aims of a research project
- **Carrier status**: test result showing that a person has inherited a recessive allele for a genetic trait without displaying that trait or showing symptoms of the disease
- **Analytical validity**: the analytical specificity and sensitivity, accuracy and precision of a test. In the context of genetic testing, how well the test predicts the presence or absence of a particular gene or genetic change
- **Clinical validity**: how well the genetic variant being analysed is related to the presence, absence or risk of a specific disease
- **Actionability**: the degree to which a finding can be used to guide (clinical) decision-making

## Footnotes

1 Example of a search string used in HumGen: “individual” AND “(result” OR “finding)” AND “(primary” OR “secondary” OR “incidental” OR “unsolicited” OR “extraneous” OR “additional)” AND “(return” OR “disclosure)” AND “(genetic” OR “genomic” OR “sequencing)” AND “research”, and PopGen: “biobank” AND “population”.
